# Mental Illness Strikes at the Heart: Impact of Psychiatric Diseases on Ventricular Ejection Fraction in Patients with Acute Coronary Syndromes

**DOI:** 10.3390/life15030340

**Published:** 2025-02-21

**Authors:** Marianna Mazza, Giorgio Veneziani, Francesco Maria Lisci, Sofia Morini, Gianandrea Traversi, Greta Sfratta, Caterina Brisi, Maria Benedetta Anesini, Francesca Bardi, Elisabetta Benini, Claudia Calderoni, Luca Chisari, Arianna Crupi, Emanuela De Chiara, Luca Lo Giudice, Luca Onori, Ilenia Sessa, Marta Balocchi, Roberto Pola, Eleonora Gaetani, Benedetta Simeoni, Francesco Franceschi, Gabriele Sani, Marcello Covino, Carlo Lai, Enrico Romagnoli, Giuseppe Marano

**Affiliations:** 1 Unit of Psychiatry, Fondazione Policlinico Universitario Agostino Gemelli IRCCS, Largo Agostino Gemelli 8, 00168 Rome, Italy; mariannamazza@hotmail.com (M.M.); fmlisci@gmail.com (F.M.L.); caterinabrisi8@gmail.com (C.B.); mbenedetta@hotmail.it (M.B.A.); dechiaraemanuela@gmail.com (E.D.C.);; 2 Department of Neurosciences, Università Cattolica del Sacro Cuore, Largo Agostino Gemelli 8, 00168 Rome, Italy; 3 Department of Dynamic and Clinical Psychology, and Health Studies, Sapienza University, Via degli Apuli 1, 00185 Rome, Italy; 4 Department of Cardiovascular Sciences, Fondazione Policlinico Universitario Agostino Gemelli IRCCS, Largo Agostino Gemelli 8, 00168 Rome, Italyenromagnoli@gmail.com (E.R.); 5 Unit of Medical Genetics, Department of Laboratory Medicine, Ospedale Isola Tiberina-Gemelli Isola, Via di Ponte Quattro Capi 39, 00186 Rome, Italy; 6 Section of Internal Medicine and Thromboembolic Diseases, Department of Internal Medicine, Fondazione Policlinico Universitario Agostino Gemelli IRCCS, Università Cattolica del Sacro Cuore, Largo Agostino Gemelli 8, 00168 Rome, Italy; 7 Department of Translational Medicine and Surgery, Fondazione Policlinico Universitario Agostino Gemelli IRCCS, Università Cattolica del Sacro Cuore, Largo Agostino Gemelli 8, 00168 Rome, Italy; 8 Unit of Internal Medicine, Cristo Re Hospital, Via delle Calasanziane 25, 00167 Rome, Italy; 9 Emergency Medicine Department, Fondazione Policlinico Universitario Agostino Gemelli IRCCS, Università Cattolica del Sacro Cuore, Largo Agostino Gemelli 8, 00168 Rome, Italy; benedetta.simeoni@policlinicogemelli.it (B.S.); marcello.covino@policlinicogemelli.it (M.C.)

**Keywords:** acute coronary syndromes, left ventricular ejection fraction, mental illnesses, cardiovascular risk, ACS, LVEF

## Abstract

Mental illnesses can have a significant impact on individuals experiencing acute coronary syndromes (ACS). Mental illnesses are associated with an increased cardiovascular risk profile and early onset of cardiovascular disease. A critical aspect of this interplay is the effect of psychiatric conditions on left ventricular ejection fraction (LVEF), a key parameter in evaluating cardiac function and predicting long-term outcomes in ACS patients. The present single-center, retrospective study investigated the associations between psychiatric conditions and cardiac function, with a focus on LVEF in ACS patients. The inclusion criteria were Italian nationality and 30 years or older. One hundred and sixty-four patients without (M_age_ = 68.8 ± 10.6, 62 females) and 161 patients with a psychiatric diagnosis (M_age_ = 68.4 ± 13.7, 63 females) were enrolled. The data collected included sociodemographic variables, psychiatric diagnoses, LVEF, ACS type (STEMI/NSTEMI), smoking status, previous interventions, and pharmacological treatments. Statistical analyses included chi-square, t-tests, ANOVAs, and ANCOVA to assess differences across groups. Findings revealed lower LVEF in patients with a psychiatric diagnosis compared to patients without a psychiatric diagnosis (*p* = 0.004, d = 0.36). Patients without a psychiatric diagnosis were associated with NSTEMI (*p* = 0.047, φ = 0.11), hypertension (*p* = 0.003, φ = −0.16), and dyslipidemia (*p* = 0.022, φ = −0.13). In contrast, patients with a psychiatric diagnosis were associated with STEMI (*p* = 0.047, φ = 0.11), neurological dysfunction (*p* = 0.014, φ = 0.14), and chronic obstructive pulmonary disease (*p* = 0.010, φ = 0.14). Among psychiatric diagnoses, anxiety disorders were associated with lower LVEF compared to substance abuse disorders (*p* = 0.012, d = −0.81). The findings underscore the complex relationship between mental illness and cardiac function, emphasising the need to integrate psychiatric evaluations into cardiology care to optimise the management of both mental and cardiovascular health. This study has several limitations, including its design, which prevents causal conclusions, and the use of convenience sampling, which limits the generalizability of the findings.

## 1. Introduction

Cardiovascular diseases (CVD) and mental illnesses are two major global health challenges, each contributing significantly to morbidity and mortality [1]. When these conditions intersect, particularly in the context of acute coronary syndromes (ACS), the complexities of patient care increase dramatically. Patients suffering from severe mental illnesses (SMI) such as schizophrenia, bipolar disorder, and major depression with psychotic symptoms or treatment-resistant depression, affecting 2–4% of the general population, often face unique barriers that can delay the diagnosis and treatment of ACS [1]. Mental illnesses impact the effectiveness of critical procedures for ACS, underscoring the urgent need for integrated care approaches to enhance outcomes for these vulnerable patients [1].

The prevalence of mental illnesses among patients with ACS is notably high. In particular, Mejìa et al. in 2023 described that patients with ACS show a prevalence of 14% of mild depression, 12% of moderate depression and 15% of high/severe depression, while the prevalence of mild anxiety was 38%, moderate anxiety was 17% and high/severe anxiety was 10% [2]. Studies have shown that individuals with SMI are at an increased risk of developing cardiovascular diseases, and in particular, the risk of cardiovascular mortality and sudden cardiac death (SCD) is five times higher in this population, with a reduced life expectancy of 15–20 years [2,3]. Furthermore, post-mortem studies failed to demonstrate a significant increase in coronary artery calcifications (CAC), even if an increased mortality rate from three to four-fold has been detected in patients with low CAC scores [3,4].

This discrepancy suggests that other factors, such as inflammation, autonomic dysfunction, and behavioural risk factors, may play a more significant role in the increased cardiovascular risk associated with mental illnesses [5]. Chronic inflammation and autonomic dysfunction, commonly observed in psychiatric conditions, can exacerbate cardiovascular issues. Additionally, behavioural factors such as medication non-adherence, smoking, and poor diet, which are more prevalent among those with mental illnesses, further elevate cardiovascular risk [5,6,7].

Acute Coronary Syndrome (ACS) encompasses a spectrum of urgent cardiac conditions, including unstable angina and myocardial infarction, which necessitate timely and effective medical interventions to prevent adverse outcomes [8]. ACS is a leading cause of morbidity and mortality worldwide, and its management has significantly evolved over the years, with an emphasis on rapid diagnosis and treatment [9]. The standard of care for ACS involves a series of time-dependent procedures, such as immediate reperfusion therapy, PCI, and the administration of antithrombotic agents [10]. These procedures are crucial for improving patient survival and minimizing myocardial damage.

The relationship between depression and ACS is likely mediated by a complex interplay of biological and behavioural mechanisms. Among these are inflammation [11], autonomic dysfunction [12], changes in platelet reactivity and endothelial function [13], neuroendocrine disturbances [14], lifestyle factors, and inherent risk factors [15]. This intricate network of interacting systems likely modulates both cardiac and neuropsychiatric processes.

A significant proportion of patients with ACS experience clinically significant depressive symptoms following a cardiac event. Approximately 10% of ACS patients are diagnosed with major depressive disorder [16]. Patients experiencing this dual diagnosis face an elevated risk of subsequent cardiovascular events, including recurrent myocardial infarction and stroke, as well as increased mortality rates [17]. Beyond these life-threatening risks, the presence of depression in ACS patients often leads to diminished physical capacity, hindering their ability to engage in daily activities and impacting their overall quality of life. The consequences of this comorbidity extend beyond the individual’s immediate health. Depression in ACS patients is further linked to difficulties in adhering to prescribed treatment regimens, including medication adherence and participation in cardiac rehabilitation programs [18]. The association between ACS and depression has been consistently observed to be associated with a higher incidence of adverse events [19]. Specifically, patients with this dual diagnosis experience increased rates of recurrent cardiovascular events and mortality, reduced quality of life [20], and greater healthcare costs [21,22].

This phenomenon has been investigated from two primary perspectives. One line of inquiry has focused on the complexity of comorbidities, examining the influence of cardiac disease severity and other ACS risk factors [23]. The other has explored the nature of depression itself, demonstrating the impact of specific symptoms and time of onset on outcomes in ACS patients [24,25]. Because of this evidence and because depression can be treated effectively, several professional societies have issued recommendations for depression screening among patients with ACS, coupled with comprehensive treatment upon detection of depression [26]. Recognizing the profound impact of depression on individuals with ACS, the American Heart Association has taken a proactive stance by issuing a Scientific Statement that explicitly identifies depression as a risk factor in patients hospitalized for ACS [27]. This designation underscores the importance of recognizing and addressing depression as an integral component of comprehensive ACS care.

However, the presence of mental illnesses can complicate the management of ACS, potentially delaying diagnosis and treatment and worsening patient outcomes [28]. Delays in diagnosis may arise from altered pain perception, reduced health-seeking behaviour, impaired symptom communication, and atypical clinical presentations, which can be further influenced by the side effects of psychotropic medications [29]. The presence of psychiatric diseases can indeed complicate the management of ACS and potentially delay diagnosis. While the pain associated with ACS is typically severe and prompts patients to seek medical evaluation, several factors related to psychiatric conditions can contribute to diagnostic delays. For instance, patients with psychiatric illnesses may experience or report atypical symptoms of ACS, such as anxiety or fatigue, which can be misattributed to their psychiatric condition rather than a cardiac issue. This can lead to an initial underestimation of the severity of their symptoms. Additionally, psychiatric disorders such as depression, anxiety, or schizophrenia may impair a patient’s ability to effectively communicate their symptoms to healthcare providers, resulting in a lack of clarity regarding the nature and severity of their condition. Moreover, patients with psychiatric conditions might face challenges in navigating the healthcare system, leading to delays in seeking or accessing appropriate medical care. This includes difficulties in recognizing the need for urgent evaluation or delays in presenting to emergency departments. Both patients and healthcare providers may also hold biases or misconceptions about the seriousness of cardiac symptoms in individuals with psychiatric disorders, which can result in the symptoms being dismissed or not investigated thoroughly. The presence of comorbid psychiatric conditions and the use of psychotropic medications can further complicate the clinical picture, making it harder to diagnose ACS promptly. For example, some medications can mask symptoms or mimic cardiac distress. By addressing these factors, healthcare providers can improve the timely diagnosis and management of ACS in patients with psychiatric illnesses, ultimately leading to better patient outcomes.

Mental illnesses, such as schizophrenia, anxiety, and major affective disorders, are prevalent among cardiac patients and have been linked to poorer health outcomes [30]. These conditions can significantly impact patient behaviour, treatment adherence, and symptom perception, potentially delaying the initiation of life-saving procedures [30].

A growing body of evidence underscores the bidirectional relationship between the mind and body [10,11]. Mental health conditions can not only be a consequence of cardiovascular disease but can also contribute to its development and progression. Psychological factors, such as stress and depression, can trigger physiological changes that increase the risk of cardiovascular events [31,32,33,34,35,36,37,38].

One critical yet not fully explored aspect of this interplay is the effect of psychiatric conditions on left ventricular ejection fraction (LVEF), a key parameter in evaluating cardiac function and predicting long-term outcomes in ACS patients. Emerging evidence suggests that psychological stress, emotional dysregulation, and neuroendocrine disturbances associated with mental illnesses can exacerbate myocardial injury, impair cardiac remodelling, and attenuate recovery of LVEF post-ACS [32]. Mental illnesses such as depression, anxiety, and post-traumatic stress disorder (PTSD) are associated with heightened sympathetic activation, hypothalamic-pituitary-adrenal (HPA) axis dysregulation, and inflammatory responses, all of which contribute to adverse cardiovascular effects [14,15,16]. Chronic stress and emotional dysregulation can lead to elevated levels of catecholamines, increased heart rate variability, and endothelial dysfunction, directly impairing myocardial contractility and exacerbating ischemic injury [36,39,40,41,42,43]. Furthermore, the neurohormonal and inflammatory cascades triggered by psychiatric conditions and psychopharmacological treatments may interfere with cardiac remodelling processes, diminishing the recovery of LVEF following myocardial insult [13,17,18,19].

Compounding these physiological mechanisms, behavioural factors linked to psychiatric illnesses, such as poor medication adherence, unhealthy lifestyle choices, and delayed medical care, can further hinder optimal cardiac recovery. Recent studies have also highlighted the potential role of shared genetic and epigenetic pathways between mental illnesses and cardiovascular dysfunction, underscoring the complex and multifaceted nature of this relationship [20,21,22,23]. Despite these insights, the associations between psychiatric conditions and LVEF remain insufficiently characterized, and understanding this dynamic is crucial for tailoring holistic treatment strategies in ACS patients [9].

Evidence from the existing literature highlights the importance of a multifaceted approach, particularly for psychiatric patients requiring time-sensitive procedures [44]. Implementing integrated care models that combine cardiovascular and mental health services can enhance outcomes [45]. This includes routine screening for mental illnesses in ACS patients and providing appropriate mental health support [46,47,48]. Educating and training healthcare providers who may not frequently encounter psychiatric conditions, as well as developing communication strategies tailored to psychiatric patients, can help overcome barriers, enhance understanding, and improve care delivery [24,25,26]. Moreover, the recent literature emphasizes the importance of psychological interventions in the management of CVDs [49,50]. Studies have shown that psychotherapy and psychoeducational programs can improve treatment adherence, reduce stress, and ultimately lead to better cardiovascular outcomes [51]. By addressing the psychological and social factors that contribute to cardiovascular disease, healthcare providers can optimize patient care and improve long-term outcomes.

The present study aimed to evaluate the differences in LVEF, sociodemographic characteristics, clinical data, previous interventions, and pharmacological treatment between cardiology patients without a psychiatric diagnosis and cardiology patients with a psychiatric diagnosis. Moreover, the present study assessed the differences in LVEF, sociodemographic characteristics, clinical data, previous interventions, and pharmacological treatment between patients with different psychiatric diagnoses. By integrating findings from psychiatry and cardiology, we seek to illuminate potential mechanisms, highlight clinical implications, and propose pathways for integrated care approaches that address both mental and cardiac health in this vulnerable population.

## 2. Materials and Methods

This is a single-centre, retrospective study conducted in a large university hospital, Fondazione Policlinico Universitario Agostino Gemelli IRCCS, located in Rome. We included patients who were consecutively admitted to the Emergency Department (ED) from 1 January 2018 to 30 December 2022 and who needed cardiological consultation. The inclusion criteria were as follows: (1) Italian nationality and (2) being 30 years or older. Exclusion criteria for this study included (1) patients with severe medical conditions unrelated to cardiology and (2) patients who refused to participate or did not provide informed consent. We examined the electronic medical records of included patients, collecting their demographic and clinical data. The present study was performed following the Strengthening the Reporting of Observational Studies in Epidemiology, the STROBE statement (see Supplementary Materials) [52]. The study was carried out adhering to the Principles of Human Rights, as were adopted by the World Medical Association at the 18th WMA General Assembly, Helsinki, Finland, June 1964, further amended by the 64th WMA General Assembly, Fortaleza, Ceará, Brazil, in October 2013. It received approval from the Fondazione Policlinico Agostino Gemelli Protocol N°: 0025817/22, ID:5121 of 3 August 2022.

### 2.1. Clinical Assessment

After collecting the sociodemographic characteristics (i.e., age and sex), clinical consultation assessed the presence of a mental illness according to the DSM-5 [28]. In addition, the presence of the following items in the clinical data was evaluated: the left ventricular ejection fraction (LVEF), the presence of associated acute myocardial infarction or without ST-segment elevation (STEMI/NSTEMI), being a smoker, the presence of previous stroke, myocardial infarction (MI), infarcts atrial fibrillation, hypertension, dyslipidemia, obesity, diabetes mellitus, malignancy, neurological dysfunction, chronic obstructive pulmonary disease, peripheral arterial disease or valvular disease, and family history of coronary artery disease. Echocardiographic parameters were measured following the current guidelines; in particular, global left ventricle ejection fraction (LVEF) was calculated from end-diastolic and end-systolic volumes computed on biplane echocardiographic images according to the Simpson method [53]. In addition, the clinical consultation assessed the presence of previous percutaneous coronary intervention (PCI), coronary artery bypass graft, pacemaker, implantable cardioverter-defibrillator, cardiac non-coronary surgery, and revascularization. Lastly, the typology of pharmacological treatment (i.e., Ezetimibe, Acenocoumarol or Coumadin or Heparin, Anticoagulants, and Antiplatelet) was collected. In our retrospective study, the diagnosis of psychiatric disorders was based on the medical records of the patients. Psychiatric diagnoses were documented in the psychiatric consultation report and made by board-certified psychiatrists using standardized diagnostic criteria, such as the Diagnostic and Statistical Manual of Mental Disorders (DSM-5).

### 2.2. Statistical Analyses

A priori power analysis (“*t*-tests: difference between two independent means (two groups)”) was conducted using G*Power 3.1.9.6 software. Following Cohen’s assumptions [54], a small to medium effect size of 0.4 (Cohen’s d), an alpha error probability of 0.05, and a power of 95% indicated that a total sample size of 328 (164 for each group) was required. Considering a possible attrition bias, to account for the possible loss of participants or incomplete data during the study, an over-recruitment of 10% was performed [55].

Descriptive analyses were performed on sociodemographic characteristics, clinical data, previous interventions, and pharmacological treatment. Moreover, normality violations were examined through Shapiro–Wilk’s test, as well as skewness and Kurtosis values. Possible differences in sociodemographic characteristics, clinical data, previous interventions, and pharmacological treatment between patients without and patients with a psychiatric diagnosis were assessed through chi-square and Welch t-tests.

To evaluate the differences between different mental illnesses, analyses of variance (ANOVAs) were conducted on age and LVEF by including the psychiatric conditions as a between-subject factor. Moreover, chi-square tests were conducted on sex, clinical data, previous interventions, and pharmacological treatment to assess differences between the psychiatric conditions.

Lastly, analysis of covariance (ANCOVA) evaluated how age, sex, and being a smoker affected the results of the ANOVA. Post hoc comparisons for the two ANOVAs and the ANCOVA were conducted using Tukey’s Honestly Significant Difference (HSD) test to correct for multiple comparisons. Levene’s test checked the assumption of the equality of variances. When Levene’s test was significant, Welch correction was applied.

To evaluate the effect sizes of the analyses, Cohen’s d was used for the independent t-tests to assess the magnitude of differences between the two groups. According to Cohen’s [54] conventions, a small effect is considered d = 0.2, a medium effect is d = 0.5, and a large effect is d = 0.8. For the chi-square tests, the phi coefficient (φ) and Cramér’s V were calculated to measure the strength of association between categorical variables, with values of φ = 0.1, 0.3, and 0.5 indicating small, medium, and large associations, respectively. The value of Cramér’s V ranges from 0 to 1, with higher values indicating stronger associations. The effect size was interpreted as follows: values between 0.00 and 0.10 indicate a weak association, 0.10 to 0.30 a moderate association, 0.30 to 0.50 a strong association, and values above 0.50 reflect a very strong association. Lastly, for the ANOVAs, eta squared (η^2^) was evaluated to quantify the proportion of variance explained by the independent variables in the model, with small, medium, and large effects corresponding to η^2^ = 0.01, 0.06, and 0.14, respectively.

All the analyses were performed using the statistical software JASP (v. 0.17.3), and for all the tests conducted, *p*-values of <0.05 were considered statistically significant.

## 3. Results

**Figure 1:**
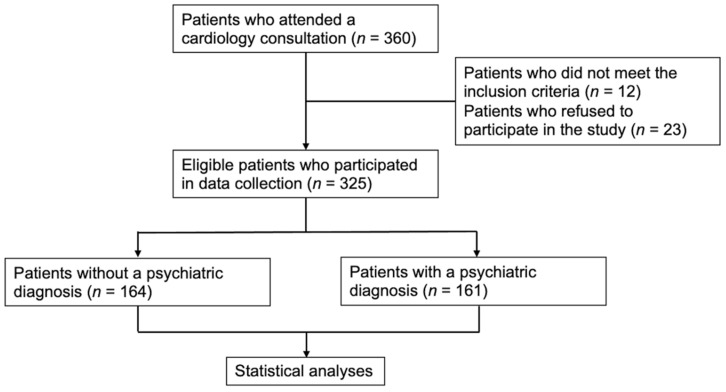
Flowchart of the patient selection process.

Three hundred and twenty-five participants signed the informed consent form and participated in the present study (Figure 1).

Table 1 shows the demographic characteristics, clinical data, previous interventions, pharmacological treatment, and differences (chi-square and t-tests) between the patients without and patients with a psychiatric diagnosis and the differences between groups. There were 164 patients without a psychiatric diagnosis (62 females; Mage = 68.8 ± 10.6) and 161 patients with a psychiatric diagnosis (63 females; Mage = 68.4 ± 13.7). Among the latter, a total of 37 patients (10 females; Mage = 70.4 ± 12.6) were diagnosed with anxiety disorder, whereas 35 patients (21 females; Mage = 69.8 ± 11.2) had a dual diagnosis of anxiety and depressive disorder. In addition, 32 patients (18 females; Mage = 72.6 ± 9.7) had a diagnosis of depressive disorder, and 35 patients (3 females; Mage = 55.6 ± 12.1) had a substance use disorder. Lastly, 22 patients had different other diagnoses, such as eating disorders, schizophrenic spectrum disorders, and neurocognitive disorders.

**Figure 2:**
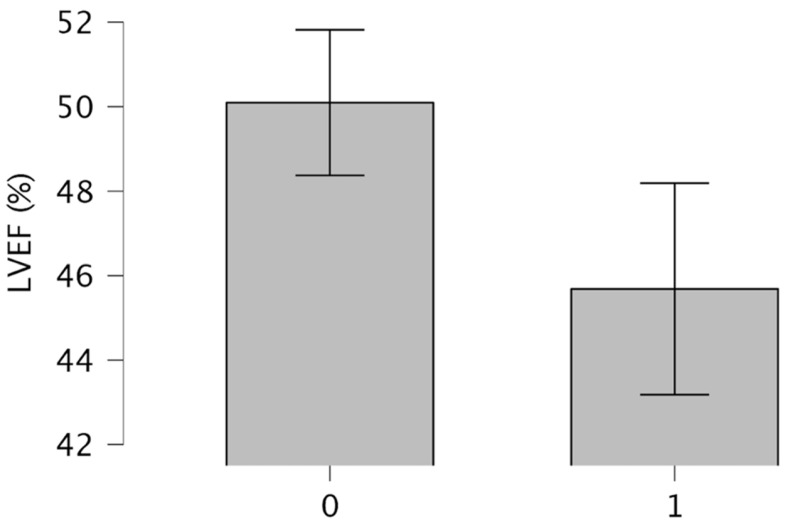
Means of LVEF between patients without and patients with a psychiatric diagnosis. Note. Patients without a psychiatric diagnosis “0”; patients with a psychiatric diagnosis “1”.

Shapiro–Wilk test revealed that the age (W = 0.98, *p* = 0.01) and LVEF (W = 0.90, *p* < 0.001) significantly deviated from normality. However, age showed a skewness of −0.29 and a Kurtosis of −0.33, whereas LVEF had a skewness of −1.33 and a Kurtosis of 2.33. Considering that their skewness was between 2 and −2 and Kurtosis was between 3 and −3, the extreme values were not very different according to normal data distribution, indicating that the distributions were only moderately asymmetrical. Regarding the clinical data, Levene’s test for equality of variances revealed a significant difference in age (F(1, 323) = 15.05, *p* < 0.001) and LVEF (F(1, 254) = 11.55, *p* < 0.001) variances between patients without and patients with a psychiatric diagnosis. Welch’s t-test showed that patients without a psychiatric diagnosis had a higher left ventricular ejection fraction (LVEF) compared to those with a psychiatric diagnosis (Table 1). Figure 2 reports the means of LVEF between patients without and patients with a psychiatric diagnosis.

Additionally, chi-square tests indicated significant associations between the group (patients without and patients with a psychiatric diagnosis) and several clinical variables (Table 1). Patients without a psychiatric diagnosis were more likely to experience NSTEMI (Adjusted standardized residuals (Adj. stzd. Res.) = 1.98), have hypertension (Adj. stdz. res. = 2.94), dyslipidemia (Adj. stdz. res. = 2.94) and were less likely to present chronic obstructive pulmonary disease (Adj. stdz. res. = 2.46) and neurological dysfunction (Adj. stdz. res. = 2.46). In contrast, patients with a psychiatric diagnosis were more likely to experience STEMI (Adj. stzd. Res. = 1.98), neurological dysfunction (Adj. stdz. res. = 2.46), and chronic obstructive pulmonary disease (Adj. stdz. res. = 2.46). At the same time, they were less likely to present hypertension (Adj. stdz. res. = 2.94) and dyslipidemia (Adj. stdz. res. = 2.94).

**Figure 3:**
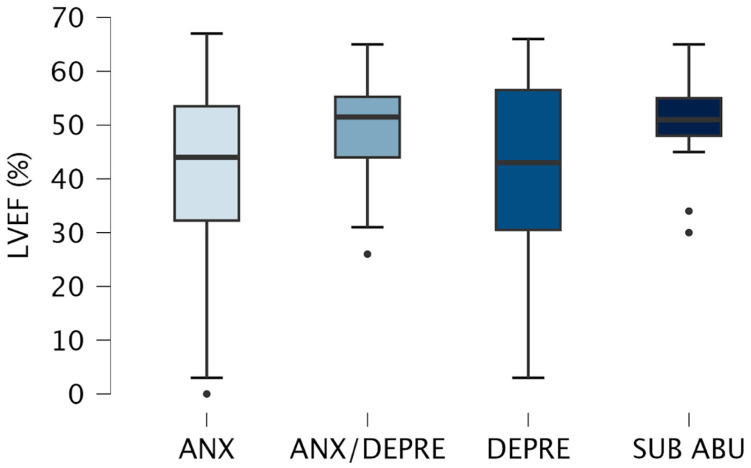
Means of LVEF across psychiatric subgroups. Note. Left Ventricular Ejection Fraction “LVEF”; Anxiety disorder group “ANX”; Dual diagnosis of anxiety and depression disorders group “ANX/DEPRE”; Depression disorder group “DEPRE”; Substance abuse disorder group “SUB ABU”.

Concerning the differences in LVEF between the psychiatric conditions (Table 2), Levene’s tests showed a significant difference (F(3, 107) = 8.27, *p* < 0.001) among patients with different psychiatric diagnoses. Figure 3 presents the means of LVEF across psychiatric subgroups.

The ANOVA showed a significant main effect, where patients with anxiety disorders had significantly lower LVEF percentages than patients with substance abuse disorder. Moreover, there were differences in age between the psychiatric conditions (Table 2), where patients with anxiety disorders had significantly higher age than patients with substance abuse disorder, patients with both anxiety and depression diagnoses had significantly higher age than patients with substance abuse disorder, and, lastly, patients with depression diagnosis had significantly higher age than patients with substance abuse disorder.

It is noteworthy that the chi-square tests conducted on differences in sociodemographic, clinical, anamnestic and treatment variables showed that in the groups of patients with a diagnosis of depression (Adj. stdz. res. = 2.51) and patients with dual diagnosis of anxiety and depression (Adj. stdz. res. = 3.19) there were significantly more females than males, while in the group of patients with substance abuse disorder (stdz. res. = 4.08) there were more males than females (χ^2^[3, 139] = 26.61, *p* < 0.001, Cramér’s V = 0.44). In addition, there were more nonsmokers than smokers in the group of patients with anxiety disorders (Adj. stdz. res. = 2.31) and patients with depressive disorders (Adj. stdz. res. = 2.50), while there were more smokers than nonsmokers in the group of patients with substance use disorder (stdz. adj. res. =6.12) (χ^2^[3, 138] = 38.05, *p* < 0.001, Cramér’s V = 0.52).

Considering the differences in age, sex, and the number of smokers between the psychiatric conditions’ groups, an ANCOVA to evaluate the differences in LVEF between the psychiatric conditions controlling for age, sex, and the number of smokers was performed. The ANCOVA results showed a significant main when controlling for the age, sex, and the number of smokers (F(3, 103) = 2.769, η^2^ = 0.075, *p* = 0.04). Post hoc comparisons did not confirm that patients with anxiety disorders (M = 39.8 ± 18.7) had significantly lower LVEF percentages than patients with substance abuse disorder (M = 51.3 ± 7.8) (p_tukey_ = 0.10, 95% CI [−21.51, −1.50]).

## 4. Discussion

This study highlights the significant interplay between mental illnesses and cardiac function in patients with ACS. By comparing the left ventricular ejection fraction (LVEF) and clinical characteristics of patients with and patients without a psychiatric diagnosis, we provide valuable insights into the impact of mental illnesses on cardiac health.

A study examining cardiac function in individuals with schizophrenia on long-term antipsychotic treatment, conducted by Andreou and colleagues [39], revealed that these individuals exhibited significantly lower LVEF compared to their healthy counterparts. This observation aligns with findings from a more recent investigation into the association between severe mental illness and the development of clinical heart failure, which also highlighted the increased risk of cardiac dysfunction in individuals with psychiatric conditions [56]. These convergent findings suggest that patients with mental illness may be at heightened risk for structural changes in the left ventricle and cardiac remodeling. Such alterations could not only predispose them to developing reduced LVEF at an earlier age but also elevate their risk of experiencing heart failure in the future. Consequently, this could lead to increased mortality rates over time. In our sample, patients without a psychiatric diagnosis exhibited significantly higher LVEF compared to patients with a psychiatric diagnosis, as observed in previous studies [29,30,57]. This finding underscores the detrimental impact of psychiatric conditions on cardiac function, likely mediated by physiological, neurohormonal, and behavioral mechanisms. Chronic inflammation (i.e., elevated IL-6 and TNF-α) and hypothalamic-pituitary-adrenal (HPA) axis dysregulation, often observed in psychiatric patients, may exacerbate myocardial injury [58,59]. Elevated catecholamine levels, systemic inflammation, and endothelial dysfunction are mechanisms contributing to reduced myocardial contractility and impaired recovery post-ACS. All studies agree in stating that screening psychiatric symptoms, such as depression and anxiety, in patients with ACS is pivotal to identifying patients who may need enhanced clinical treatment and support and more frequent follow-up based on a multidisciplinary approach. However, limited research has specifically examined the impact of pre-existing psychiatric conditions on ACS presentation and clinical outcomes.

The existing literature supports the role of chronic systemic inflammation, dysregulation of the hypothalamic-pituitary-adrenal (HPA) axis, and autonomic dysfunction in contributing to adverse cardiac remodeling and impaired myocardial function in psychiatric patients [60]. Elevated levels of pro-inflammatory cytokines such as interleukin-6 (IL-6) and tumor necrosis factor-alpha (TNF-α) are frequently observed in individuals with psychiatric disorders [61]. These cytokines are known to promote inflammation and oxidative stress, which can lead to endothelial dysfunction, atherosclerosis, and ultimately adverse cardiac remodeling [62]. Dysregulation of the HPA axis, often seen in conditions such as depression and anxiety, results in sustained elevated cortisol levels [63]. Chronic exposure to high cortisol can cause cardiomyocyte apoptosis, myocardial fibrosis, and impaired cardiac contractility, further exacerbating cardiac dysfunction [64]. Moreover, autonomic dysfunction, characterized by reduced heart rate variability (HRV) and increased sympathetic activation, is commonly present in psychiatric patients [65]. Reduced HRV is associated with a higher risk of arrhythmias and sudden cardiac death, while increased sympathetic activity can lead to hypertension, increased myocardial oxygen demand, and adverse structural changes in the heart [66]. Studies have shown that these autonomic alterations can significantly impair myocardial function and contribute to the progression of heart failure [67,68]. Consequently, the interplay between chronic inflammation, HPA axis dysregulation, and autonomic dysfunction highlights the complex pathophysiological mechanisms through which psychiatric disorders can detrimentally affect cardiac health [60]. Further research is necessary to elucidate these mechanisms and develop targeted therapeutic strategies to mitigate the cardiovascular risk in psychiatric patients. Serotonin’s influence on cardiovascular control involves complex interactions with hormonal and autonomic nervous system activity. Beyond their mood-stabilizing effects, medications impacting brain serotonin levels can modulate circulating adrenocorticotropic hormone (ACTH) and glucocorticoid concentrations [69]. These hormonal alterations likely stem from serotonergic input to corticotropin-releasing factor (CRF)-producing cells within the hypothalamic paraventricular nucleus [70]. Hypothalamic serotonin activity appears to modulate hormonal responses to stress [71]. For example, antidepressant therapy can normalize the disrupted cortisol feedback mechanism within the hypothalamic-pituitary-adrenal (HPA) axis observed in some individuals with depression [72]. Conversely, disruption of hypothalamic serotonin pathways can amplify the suppressive effects of dexamethasone on stress-induced adrenocortical activity [73]. The hypothalamic paraventricular nucleus, a target of serotonergic projections, influences both sympathetic and parasympathetic activity via connections to the spinal cord, rostral ventrolateral medulla, and dorsal vagal complex [74]. Regarding serotonin’s role in cardiovascular processes, studies indicate heightened sensitivity to its vasoconstrictive effects in vessels compromised by hypertension or atherosclerosis [75]. Furthermore, research has observed altered central nervous system serotonin levels in rodents experiencing myocardial ischemia [73]. Genetic studies have linked a specific serotonin transporter polymorphism to increased myocardial infarction risk in men post-heart attack [76]. Platelet dysfunction, partially modulated by serotonin and implicated in cardiovascular disease development, has also been observed in depression [77]. Collectively, these observations suggest a potential connection between depressive disorders and the neuroendocrine and autonomic systems, ultimately impacting cardiovascular health. Our data indicate a significant association between psychiatric conditions and the type of ACS, with patients without a psychiatric diagnosis being more frequently associated with non-ST-elevation myocardial infarction (NSTEMI), while patients with a psychiatric diagnosis were more likely to experience ST-elevation myocardial infarction (STEMI). This result is coherent with previous research that highlighted how mental illnesses can increase the risk of ACS, such as STEMI [78]. Indeed, mental illnesses may negatively impact health behaviors, including adherence to medication, exercise, and diet, which are key factors in cardiovascular risk management, emphasizing the importance of integrated care models that address both mental and physical health [47]. Indeed, it is acknowledged that physical illnesses, mainly cardiovascular diseases, substantially contribute to the high mortality rates in patients with severe mental illnesses [79]. Promoting attention to psychological states and improving psychological distress may enhance symptom appraisal and facilitate timely care-seeking to avert further deterioration.

Individuals with severe mental illness, including psychotic spectrum disorders, experience a significantly higher risk of cardiometabolic disorders compared to the general population. This disparity results in a 2- to 3-fold increase in mortality, primarily due to cardiovascular disease, and a potential reduction in life expectancy of up to 20% [80]. This elevated CVD risk is driven by a complex interplay of factors, including unhealthy lifestyle behaviors, the metabolic side effects of psychotropic medications, and the physiological impacts of chronic stress [81]. Unhealthy dietary patterns, physical inactivity, tobacco use, and the use of antipsychotic medications are among the specific contributors to this increased risk. It is crucial to emphasize that many CVD-related risk factors are modifiable through lifestyle adjustments and, when indicated, referral for appropriate treatment interventions. Interestingly, in this study, patients without a psychiatric diagnosis had a higher prevalence of hypertension and dyslipidemia compared to patients with a psychiatric diagnosis. While these conditions are traditional cardiovascular risk factors, their lower prevalence in patients with a psychiatric diagnosis might reflect underdiagnosis or insufficient management of comorbidities in this group. Alternatively, the cardiovascular burden in patients with a psychiatric diagnosis might arise from distinct mechanisms, including autonomic dysregulation and chronic systemic inflammation. Neurological dysfunction and chronic obstructive pulmonary disease (COPD) were more common among patients with a psychiatric diagnosis, suggesting a broader spectrum of systemic comorbidities. These comorbidities increase disease burden and may complicate the treatment of the combined disorders, substantially impair quality of life, and are under-treated [31]. Neurological dysfunction may reflect shared pathophysiological pathways between psychiatric and cardiovascular diseases, while COPD’s prevalence may be related to higher smoking rates in psychiatric populations [32].

The analysis of psychiatric diagnoses in our sample of patients revealed further complexities. Patients with anxiety disorders exhibited the lowest LVEF among psychiatric subgroups. Anxiety’s heightened sympathetic activation and systemic inflammatory response likely exacerbate cardiac remodeling and impair myocardial recovery. It has already been outlined that mental, but not exercise, stress-induced left ventricular ejection fraction change significantly predicts the risk of future adverse cardiovascular events [33]. Conversely, patients with substance use disorders, typically younger, demonstrated comparatively preserved LVEF, potentially due to fewer cumulative cardiovascular insults. It is noteworthy that the present study showed a significant main effect of psychiatric conditions on LVEF, but no significant post-hoc differences were found after controlling for age, sex, and the number of smokers between the groups of patients with a psychiatric diagnosis. In this regard, smoking is a well-established risk factor for cardiovascular diseases [82], and previous research found age and sex as pivotal factors in cardiovascular outcomes [83,84,85]. Consistently, although mental illnesses would impact cardiac function, the present study’s findings underscore the importance of considering the complexity of the relationship between psychiatric conditions and cardiac function, considering also the indirect factors (e.g., lifestyle factors) that could influence cardiac function.

Anxiety and depression were associated with older age compared to substance use disorders. The older age of these patients may contribute to cumulative cardiovascular damage, compounding the deleterious effects of psychiatric conditions on cardiac health. This finding highlights the need for age-specific interventions in managing psychiatric comorbidities in ACS.

The associations between psychiatric illnesses and cardiovascular dysfunction are evident in this study. Beyond behavioral factors like smoking and medication non-adherence, psychiatric conditions contribute to adverse outcomes through neurohormonal dysregulation and chronic inflammation [86,87]. Psychopharmacological treatments, particularly antipsychotics, may further elevate cardiovascular risk by inducing metabolic disturbances. Shared genetic and epigenetic pathways between psychiatric and cardiovascular diseases may also play a critical role in this complex interaction [34,35].

Integrated care approaches are essential for managing patients with concurrent psychiatric and cardiac conditions. Routine screening for mental illnesses in ACS patients and providing targeted mental health interventions can mitigate cardiovascular risks. Multidisciplinary teams—comprising cardiologists, psychiatrists, and psychologists—are vital for delivering holistic care. Psychological interventions such as cognitive-behavioral therapy and psychoeducation can improve treatment adherence and lifestyle modifications, reducing cardiovascular complications. Early psychological counselling post-ACS may help prevent stress-related cardiac dysfunction, enhancing long-term recovery. Educating healthcare providers about the intersection of psychiatric and cardiovascular diseases is crucial for improving outcomes [36].

This study provides valuable insights despite several limitations that must be addressed. The primary limitation is its design, which does not allow for establishing causal relationships between variables. These constraints necessitate a cautious interpretation of the present findings and underscore the need for future prospective investigations to more robustly determine causal relationships. Additionally, the sampling method used introduces potential selection bias, as findings based on convenience samples may lack generalizability to the broader population from which the sample was drawn. Consequently, caution is required when interpreting these results in a wider context. The present study did not consider important details of the psychiatric treatment or the severity and duration of the disorders. While it is established that the management of conditions such as depression and anxiety may have an impact on disease outcomes following ACS, the absence of data regarding the psychiatric treatment received by patients did not allow the evaluation of their potential effects [47,88,89]. Future studies should consider incorporating information on psychiatric treatment to understand its role in shaping cardiovascular outcomes in patients with coexisting mental illnesses. To strengthen future research, longitudinal study designs and predictive statistical models should be implemented to explore better the intricate relationship between mental illnesses and cardiovascular diseases. Moreover, investigating additional factors, such as the severity of psychopathological symptoms, could offer a more comprehensive understanding of the influence of psychiatric conditions on cardiac function.

## 5. Conclusions

This study elucidates the profound impact of psychiatric illnesses on cardiac function in ACS patients, particularly regarding LVEF. Highlighting the interplay between mental and cardiac health underscores the necessity of integrated care models. The co-occurrence of depression and cardiovascular disease presents a critical public health challenge, emphasizing the importance of elucidating the underlying mechanisms to develop effective interventions.

Interdisciplinary collaboration is considered a cornerstone of comprehensive patient care. The synergy between clinical and experimental science offers a powerful approach to understanding mood and cardiovascular regulation in humans. Such collaborations can produce translational findings that facilitate research exploring the integrated roles of behaviour, physiology, and brain function in mood and cardiovascular disorders. Tailored interventions and heightened awareness among healthcare providers can bridge the gap between psychiatry and cardiology, improving outcomes for this high-risk group.

Evidence-based treatments for mental illness, particularly depression and anxiety, in patients with ACS include psychological interventions such as cognitive-behavioral therapy (CBT) and mindfulness-based stress reduction (MBSR) [90]. Recent studies have highlighted the effectiveness of positive psychology interventions, which focus on enhancing positive psychological constructs and have been associated with superior outcomes in cardiac patients [91]. Furthermore, psychosocial interventions that address both depression and social support have been found to improve health outcomes in ACS patients. A systematic review found that psychosocial interventions resulted in modest reductions in depressive symptoms and improvements in social support [92]. Emotion-focused coping strategies at the time of the cardiac event have been detected as a reliable psychological predictor of disease severity at a three-month follow-up, suggesting that emotional coping may be a fruitful target for psychological treatments in patients with ACS, contributing to improving LVEF and cardiac outcome [93]. Even though greater interest has been given to the psychological-integrated model, further studies need to be performed. Additionally, aerobic exercise and cardiac rehabilitation programs, which often include psychological support, have been shown to improve mental health-related quality of life in ACS patients [92]. Nevertheless, the observational design and reliance on convenience sampling may limit the broader applicability of the results. Factors such as geographic location, socioeconomic background, or specific clinical characteristics of the sample could affect the results, reducing the possibility of generalizing them to all patients with coronary syndromes or mental illnesses. Future multi-center studies should employ longitudinal designs to establish causal relationships between psychiatric conditions and cardiac outcomes, clarifying the temporal dynamics between mental health and cardiovascular disease and allowing for more conclusions regarding causality.

## Figures and Tables

**Table 1:** Demographic characteristics, clinical data, previous interventions, and pharmacological treatment of patients without and patients with a psychiatric diagnosis, as well as differences (chi-square and t-tests) between groups.

Variable	Patients		χ^2^/t (*p*-Value [CI 95%], Effect Size)	Total Sample (*N* = 325)
	Without a PD (*N* = 164)	With a PD (*N* = 161)		
Sociodemographic characteristics				
Age (M ± SD)	68.8 ± 10.6	68.4 ± 13.7		68.6 ± 12.3
Sex				
Male	102	98		200
Female	62	63		125
Missing	0	0		0
Clinical data				
LVEF (M_%_ ± SD)	50.1 ± 9.8	45.7 ± 14.4	2.87 (0.004 [1.38, 7.44], d = 0.36) ^a^	
Missing	38	31		
STEMI/NSTEMI			3.93 (0.047 [0.01, 0.89], φ = 0.11)	
NSTEMI	87	67		154
STEMI	72	87		159
Missing	5	7		12
Current smoker				
No	107	93		200
Yes	54	64		118
Missing	3	4		7
Family history of coronary artery disease				
No	110	117		227
Yes	50	41		91
Missing	4	3		7
Any prior MI				
No	120	122		242
Yes	40	36		76
Missing	4	3		7
Peripheral Artery Disease				
No	138	136		274
Yes	22	22		44
Missing	4	3		7
Valve disease				
No	137	139		276
Yes	22	17		39
Missing	5	5		10
Prior Stroke				
No	143	143		286
Yes	16	15		31
Missing	5	3		8
Atrial fibrillation				
No	138	137		275
Yes	22	21		43
Missing	4	3		7
Hypertension			8.67 (0.003 [−1.36, −0.26], φ = −0.16)	
No	25	46		71
Yes	137	112		249
Missing	2	3		5
Dyslipidemia			5.22 (0.022 [−0.96, 0.07], φ = −0.13)	
No	67	85		152
Yes	95	72		167
Missing	2	4		6
Obesity				
No	122	129		251
Yes	26	20		46
Missing	16	12		28
Malignancy				
No	140	134		274
Yes	21	22		43
Missing	3	5		8
Neurological dysfunction			6.06 (0.014 [0.16, 1.61], φ = 0.14)	
No	148	132		280
Yes	12	26		38
Missing	4	3		7
Diabetes Mellitus				
No	109	111		220
Yes	51	47		98
Missing	4	3		7
Chronic obstructive pulmonary disease				
No	153	138	6.59 (0.010 [0.22, 1.91], φ = 0.14)	291
Yes	8	21		29
Missing	3	2		5
Previous interventions				
PCI				
No	116	120		236
Yes	44	36		80
Missing	4	5		9
Coronary artery bypass graft				
No	152	151		303
Yes	8	5		13
Missing	4	5		9
Pacemaker				
No	158	152		310
Yes	2	5		7
Missing	4	4		8
Implantable cardioverter-defibrillator				
No	146	149		295
Yes	6	6		12
Missing	12	6		18
Cardiac non coronary surgery				
No	157	153		310
Yes	3	2		5
Missing	4	6		10
Revascularization				
No	117	118		235
Yes	43	38		81
Missing	4	5		9
Pharmacological treatment				
Ezetimibe				
No	138	146		284
Yes	12	11		23
Missing	14	4		18
Sintrom or Coumadin or Eparina				
No	140	140		280
Yes	10	18		28
Missing	14	3		17
Anticoagulants				
No	136	142		278
Yes	13	16		29
Missing	15	3		18
Antiplatelet				
No	13	13		26
Yes	137	144		281
Missing	14	4		18

Note. Psychiatric Diagnosis “PD”; ^a^ Welch *t*-test; Mean “M”; Standard Deviation “SD”; Confidence Intervals “CI”; Cohen’s d “d”; Phi coefficient “φ”; Left Ventricular Ejection Fraction “LVEF”; Myocardial Infarction “MI”; Percutaneous Coronary Intervention “PCI”; Acute Myocardial Infarction with ST-segment elevation “STEMI”; Acute Myocardial Infarction without ST-segment elevation; “NSTEMI”.

**Table 2:** ANOVA (Welch correction) between the Psychiatric conditions (Anxiety, Anxiety and Depression, Depression, and Substance abuse disorders) on left ventricular ejection fraction (LVEF) and age.

DV	Psychiatric Conditions (Number of Patients; Mean ± SD)				Sum of Squares (res)	df (res)	Mean Square (res)	F (*p*-Value, Effect Size)	Post-Hoc (*p*-Value, [CI 95%])
LVEF (%)	ANX (30; 39.8 ± 18.7)	ANX/DEPRE (28; 49.0 ± 9.5)	DEPRE (24; 42.0 ± 17.7)	SUB ABUSE (29; 51.3 ± 7.8)	2.603 (21.410)	3.0 (55.0)	867 (389)	4.434 (0.007, η^2^ = 0.11)	SUB ABU > ANX (0.012, [−21.13, −1.89]) ^a^
Age	ANX (37; 70.4 ± 12.6)	ANX/DEPRE (35; 69.8 ± 11.2)	DEPRE (32; 72.6 ± 9.7)	SUB ABUSE (35; 55.6 ± 12.1)	6.242 (17.877)	3.0 (75.0)	2.080 (238)	15.086 (<0.001, η^2^ = 0.26)	SUB ABU < ANX (<0.001, [7.74, 21.86]) ^a^ SUB ABU < ANX/DEPRE (<0.001, [7.04, 21.36]) ^a^ SUB ABU < DEPRE (<0.001, [9.64, 24.28]) ^a^

Note. ^a^ Tukey corrected; Dependent variable “DV”; Left Ventricular Ejection Fraction (LVEF); Standard Deviation “SD”; Anxiety disorder “ANX”; Dual diagnosis of anxiety and depression disorders “ANX/DEPRE”; Depression disorder “DEPRE”; Substance abuse disorder “SUB ABUSE”; Residuals “res”.

## Data Availability

Data are available upon request from the corresponding author.

## Supplementary Materials

The following supporting information can be downloaded at https://www.mdpi.com/article/10.3390/life15030340/s1. STROBE Statement—Checklist of items that should be included in reports of cross-sectional studies
